# Molecular Imaging of Stem Cell Transplantation for Liver Diseases: Monitoring, Clinical Translation, and Theranostics

**DOI:** 10.1155/2016/4058656

**Published:** 2016-12-14

**Authors:** Ping Wang, Francesco Petrella, Luca Nicosia, Massimo Bellomi, Stefania Rizzo

**Affiliations:** ^1^Molecular Imaging Laboratory, MGH/MIT/HMS Athinoula A. Martinos Center for Biomedical Imaging, Department of Radiology, Massachusetts General Hospital, Harvard Medical School, Boston, MA 02129, USA; ^2^Department of Thoracic Surgery, European Institute of Oncology, Via Ripamonti 435, 20141 Milan, Italy; ^3^Postgraduation School in Radiodiagnostics, Università degli Studi di Milano, Via Festa del Perdono 7, 20122 Milan, Italy; ^4^Department of Radiology, European Institute of Oncology, Via Ripamonti 435, 20141 Milan, Italy; ^5^Department of Oncology, University of Milan, Via Festa del Perdono 7, 20142 Milan, Italy

## Abstract

Stem cell transplantation has been investigated to rescue experimental liver failure and is promising to offer an alternative therapy to liver transplantation for liver diseases treatment. Several clinical studies in this field have been carried out, but the therapeutic benefit of this treatment is still controversial. A major obstacle to developing stem cell therapies in clinic is being able to visualize the cells in vivo. Imaging modalities allow optimization of delivery, detecting cell survival and functionality by in vivo monitoring these transplanted graft cells. Moreover, theranostic imaging is a brand new field that utilizes nanometer-scale materials to glean diagnostic insight for simultaneous treatment, which is very promising to improve stem cell-based therapy for treatment of liver diseases. The aim of this review was to summarize the various imaging tools that have been explored with advanced molecular imaging probes. We also outline some recent progress of preclinical and clinical studies of liver stem cells transplantation. Finally, we discuss theranostic imaging for stem cells transplantation for liver dysfunction and future opportunities afforded by theranostic imaging.

## 1. Introduction

Acute liver failure and cirrhosis result from a variety of acute or chronic hepatic injuries [1, 2]. Up till now, liver transplantation has been considered as the primary treatment for acute liver failure and cirrhosis and various end-stage liver diseases. However, this procedure is hindered by the lack of donor organs, technical difficulties, complications associated with immune rejection, the requirement for lifelong immunosuppression, and financial considerations [3–5]. Therefore, to develop novel treatments that can either be effective curative and/or affect the underlying pathophysiology of the liver disease is urgently needed.

Stem cell transplantation has been suggested as an effective alternate approach for liver dysfunction [6–8]. For maximal efficacy, these therapies require transplanted cell delivery to targeted tissues followed by successful cell engraftment. So far, numbers of clinic trials of stem cells transplantation for liver dysfunction have been carried out. By September 2016, more than 200 clinical trials had been registered when ClinicalTrials.gov was searched for the terms “stem cells AND liver diseases”. Among these clinical studies, some have indicated to be well tolerated and safe and confer beneficial effects in patients with liver failure, by enhancing liver function and reducing ascites and overall mortality without any major side effects [9–13]. However, the benefits of this emerging therapy for liver dysfunction in recent finished clinical trials are still conflicting. Some studies demonstrated there was no significant therapeutic effect to liver dysfunction [14]. Other concerns and critical issues remain unanswered regarding the long-term safety [9, 10, 12, 15–22]. Therefore, several meta-analyses have concluded that controversies remain in this rapidly developing field [23–26].

Meanwhile, several critical issues in clinical protocols require further investigation, such as the optimal type of cell types, the optimal therapeutic timing, the most effective stem cells amount, the best route of administration, and the primary endpoints. One thing is clear that there is an unmet clinical need to monitor the transplanted stem cells in vivo. Hence, in vivo visualization of transplanted stem cells with a robust, quantitative imaging method is essential for the monitoring of cell implantation, homing, and differentiation. The purpose of this current review is to summarize the latest developments of the various imaging modalities dedicated to monitor stem transplantation for liver dysfunctions; preclinic and clinic studies are emphasized; in addition, theranostic imaging and their future applications are also highlighted.

## 2. Imaging Methods to Monitor Transplanted Stem Cells in Animal Models

Noninvasive tracking of stem cells could facilitate its clinical translation. So far all major imaging modalities have been introduced to monitor transplanted stem cells for liver diseases. Numbers of animal studies have demonstrated that imaging modalities are essential for developing efficacious cell therapies for liver damage animal models.

### 2.1. Optical Imaging

Optical imaging, mainly based on retroviral vectors or enzymes to express fluorescent proteins, includes fluorescence imaging and bioluminescence imaging (BLI). Both these imaging methods have been tested for in vivo monitoring transplanted stem cells in liver.

Yukawa et al. investigated whether quantum dots (QDs) labeling using octa-arginine peptide (R8) for adipose tissue-derived stem cells (ASCs) could be applied for in vivo fluorescence imaging in mice with acute liver failure. The results demonstrated that heparin was effective in increasing the accumulation of transplanted ASCs in the liver using this imaging technology [27]. In another study, Akhan et al. conjugated a novel polymer based water-dispersible nanoparticles (CPN) with improved photostability, high fluorescent quantum yield, and noncytotoxicity compared to conventional dyes and quantum dots. The results showed that the labeled mesenchymal stem cells (MSCs) migrated to the liver and retained their labels in an in vivo liver regeneration model. These studies demonstrated that the utilization of florescence labeling could be a promising tool for the tracking of stem cells transplanted in liver to understand differentiation and homing mechanisms [28]. Wang et al. investigated upconversion nanoparticles- (UCNPs-) labeled mouse MSCs intravenous transplanted into mice and imaged using an in vivo upconversion luminescence (UCL) imaging system, observing the translocation of MSCs from lung where they initially accumulated, to liver [29]. Li et al. compared three-delivery routes for the MSCs transplantation to liver, including inferior vena cava (IVC), the superior mesenteric vein (SMV), and intrahepatic (IH) injection using in vivo BLI. Results showed that MSCs were firstly trapped inside the lungs, and no detectable homing to the liver and other organs was observed after IVC infusion; after SMV infusion, MSCs were dispersedly distributed and stayed as long as 7-day posttransplantation in the liver. By IH injection, MSCs distribution was only localized in the injection region of the liver [30]. In another study reported by the same group, Liu et al. evaluated survival of transplanted human MSCs in the mice liver after intrahepatic transplantation and the role of intrahepatic natural killer (NK) cells by in vivo BLI. A gradual decline in bioluminescent signals from transplanted MSCs in liver was observed. Interestingly, compared to the control group, the survival time and retention of intrahepatic MSCs decreased more rapidly in the NK-activated group, which indicated that activated NK cells potentially accelerated the rejection of transplanted MSCs [31].

### 2.2. Nuclear Imaging

Wu et al. investigated the biodistribution of human placental deciduas basalis derived mesenchymal stem cells (PDB-MSCs) in nude mice after intravenous injection by carbon radioisotope labeling thymidine ((14) C-TdR), which was able to incorporate into new DNA strands during cell replication. Labeled PDB-MSCs grafts were found mainly in the lung, liver, spleen, stomach, and left femur of the recipient animals at the whole observation period. This work demonstrated that (14) C-TdR labeling did not alter the biological characteristics of human placental MSCs and that this labeling method had potential to be used to quantify the transplanted cells in preclinical studies [32].

### 2.3. Magnetic Resonance Imaging (MRI)

Magnetic resonance imaging provides high-resolution (ranging from 50 *µ*m in animals up to 300 *µ*m in whole body clinical scanners) images capable of tracking stem cells in vivo without invasiveness and without the use of ionizing radiation. Stem cells need to be prelabeled with contrast probes for cell tracking studies by MRI. The probes for MRI noninvasively identification and tracking of transplanted cells include negative contrast agents, as superparamagnetic iron-oxide (SPIO) and ultrasmall superparamagnetic iron-oxide (USPIO) particles, and positive contrast agents, such as gadolinium. They both have been tested in small animal models for stem cells transplantation studies.

An emerging approach to visualizing stem cells by MR is to label the stem cells with perfluorocarbon (PFC) nanoemulsions, which can be detected with 19Fluorine MRI.

#### 2.3.1. SPIO

Bos et al. evaluated in vivo tracking of intravascularly injected SPIO labeled MSCs using a conventional 1.5 T MR imaging system. After labeled MSC injection, damaged liver had diffuse granular appearance. Cells were detected for up to 12 days in liver. They concluded that MRI could in vivo monitor intravascularly administered SPIO labeled MSCs in liver [33]. Arbab et al. developed a method of delivering SPIO labeled MSCs to a targeted area in an animal model by applying an external magnet. SPIO labeled MSCs were injected intravenously to animals, and an external magnet was placed over the liver area of the animals. MRI signal intensity (SI) changes in the liver were monitored at different time points using MRI. Interestingly, SI decrease in the liver after injection of MSCs was greater in rats with external magnets and returned gradually to that of control rat livers at approximately day 29. The results proved that the external magnets retained the labeled MSCs in the region of interest [34]. Cai et al. evaluated in vivo MRI for tracking the SPIO labeled MSCs transplanted into rat liver through hepatic arterial infusion. The labeled stem cells in liver could be detected and monitored in vivo with a 1.5 T clinical MR scanner for up to 7 days after transplantation [35]. Ju et al. tracked intrasplenically transplanted MSCs labeled with SPIO by using MRI in vivo in a liver damaged rat model. These studies demonstrated that MSCs could be effectively labeled with approximately 100% efficiency. Migration of transplanted labeled cells to the liver was successfully documented with in vivo MRI [36, 37]. Choi et al. labeled human MSCs with SPIO and green fluorescence protein (GFP). Labeled MSCs were transplanted into the portal veins of immunosuppressed, hepatic-damaged rat models. Serial MR imaging showed signal loss in the liver of transplanted cells in the early period of transplantation [38]. Zhong et al. assessed SPIO labeled MSCs that were transplanted via the portal vein to rats with hepatic fibrosis using MRI. T2^∗^-weighted MR imaging indicated lower signal in liver [39]. Kim et al. used a fluorescent magnetic nanoparticle (MNP), which had both magnetic and optical features, containing a ferritin core and a silica shell, and the inner portion of silica shell filled fluorescent materials for stem cell tracking studies. Intrasplenically transplanted MSCs labeled with MNP in liver cirrhosis rat model were monitored with 3T MR scanner. The results showed that the liver-to-muscle contrast-to-noise ratios at 3 and 5 h after cell transplantation was significantly lower than that of preinjection group [40].

#### 2.3.2. Gadolinium Based Probes

Shuai et al. transfected human umbilical cord derived MSCs with gadolinium-diethylenetriaminepentaacetic acid (Gd-DTPA) for in vitro MRI. The result showed that minimum 5 × 10^4^ Gd-DTPA-labeled stem cells could be detected with MRI in vitro. The trace duration time was about 12 days without compromising either cell viability or cell differentiability [41]. The same group also determined double labeling of MSCs with Gd-DTPA and fluorescent dye PKH26. Gd-DTPA and PKH26 were used to label the stem cells. MRI and fluorescence microscopy were used to detect the double-labeled MSCs. Their in vitro studies demonstrated that the proliferation and differentiation abilities of MSCs were not affected by double labeling using Gd-DTPA and PKH26 [42]. Furthermore, Pan et al. assessed tracking Gd-DTPA and PKH26-labeled human umbilical cord MSCs using MRI in vivo. The T1 values and signal intensity on T1-weighted imaging of labeled cells were significantly higher than the control. The signal intensity on T1-weighted imaging of labeled cells was retained over 14 days in vivo [43]. The results demonstrated that this labeling method was reliable and efficient; the in vivo studies of transplanted cells in liver using this labeling method need to be further investigated.

#### 2.3.3. Perfluorocarbon Based MRI

Another technique under evaluation for imaging injected stem cells in vitro and in vivo is based on perfluorocarbon (PFC) formulations, whose advantage is the high specificity due to the virtual absence of fluor from the body. The fluorine signal can be accurately quantified from the MR images [44].

An emerging approach to imaging stem cells by MRI is to label the stem cells with perfluorocarbon nanoemulsions, which can be detected with 19F MRI [45–47]. The 19F nucleus is particularly suitable for labeling as its relative MR sensitivity is only 17% less than that of 1H. Since the level of background 19F signal in host tissue is virtually absent [45], overlaying the 19F image on a conventional anatomical image allows for unambiguous, quantitative tracking of labeled cells in vivo.

A number of promising findings have been reported for the use of 19F labeling, including (1) intracellular labeling; (2) the lack of cellular toxicity; (3) the stability of cell labeling up to 21 days postlabeling; (4) positive signal with no background observed with 19F MRI; (5) biodistribution visualized by overlaying a 19F image of labeled cells on a 1H anatomical image; (6) determination of the local concentration of labeled cells by quantitative 19F MRI [48].

### 2.4. Emerging Imaging Modality: Magnetic Particle Imaging (MPI)

MPI is noninvasive and offers near-ideal image contrast, depth penetration, high sensitivity, and superb quantitativeness for single-cell tracking. Unlike MRI that applies magnetic resonance principles, MPI directly detects the iron-oxide nanoparticle-tagged cells using magnetic fields. These properties make MPI both ultrasensitive and linearly quantitative and very promising for monitoring transplanted cells in vivo. Conolly group from UC Berkeley firstly utilized MPI to monitor transplanted stem cells in vivo in animal model. Their first MPI cell tracking study demonstrated 200-cell detection limit in vitro and in vivo monitoring of graft clearance over 87 days in a rat model [49]. In a more recent study, this group imaged the intravenously transplanted MSCs using MPI. The results showed that labeled MSCs immediately entrapped in lung tissue posttransplantation and then relocated to the liver within one day. Longitudinal MPI-CT imaging demonstrated a clearance half-life of MSC iron-oxide labels in the liver at 4.6 days [50]. These first in vivo MPI results indicate that MPI offers strong utility for quantitating the transplanted stem cells labeled using SPIO.

## 3. Preclinic Imaging of Large Animal Models and Clinical Applications

Although stem cell transplantation based therapies have been clinically applied to patients, the fate of graft cells remains unknown. Small animal studies have demonstrated the feasibility and important role of in vivo imaging technologies for in vivo monitoring of transplanted stem cells. The next step is to conduct preclinic studies using large animals that are necessary for translation of these cutting edge imaging technologies to clinic.

### 3.1. Preclinic Imaging of Transplanted Stem Cells in Liver with Large Animal Models

Ito-Fujishiro et al. evaluated track implanted peripheral blood mononuclear cells labeled with SPIO on days 0 and 7 after intravenous injection into a cynomolgus monkey using a 3T MRI scanner. Labeled cells were visualized in the liver on MR images T2-weighted sequences [51]. Shi et al. investigated tracking of SPIO labeled MSCs after intraportal transplantation to swine models of acute liver injury. Results showed that signal intensity loss in the livers of swine models by SPIO labeling on the T2^*∗*^WI sequence persisted until 2 weeks after transplantation [52]. Spriet et al. compared the cell distribution and quantification shortly after intraportal vein, systemic intravenous and splenic injections of technetium-99m- (99mTc-) hexamethyl-propylene amine oxime (HMPAO) labeled MSCs in healthy beagle dogs. Scintigraphic images were obtained using gamma camera after cell transplantation to target the liver, which showed that, after the portal injection, diffuse homogeneous high uptake was observed through the liver; the systemic intravenous injection resulted in cells trapped in the lungs, whereas splenic injection led to MSCs mild splenic retention and homogeneous diffuse hepatic uptake [53].

### 3.2. The First Clinical Applications of Molecular Imaging on Stem Cells Transplantation for Liver Diseases

Gholamrezanezhad et al. investigated the biodistribution of autologous 111In-oxine labeled MSCs, after peripheral infusion in four cirrhotic patients was investigated using SPECT imaging. After intravenous infusion, the radioactivity was first observed to accumulate in the lungs of recipients. Then the radioactivity gradually increased in the liver and spleen in these four patients. Radiolabeled MSCs relocalized in liver and spleen: radioactivity decreased from 33.5% to 2% in lung and increased from 2% to 42% in spleen. This first clinic imaging study demonstrated that cell labeling with 111In-oxine was a suitable tool for tracking peripheral vein infused MSC distribution [54]. In a more recent case report study, Defresne et al. assessed biodistribution of 111-Indium DTPA labeled adult derived human liver stem cells (ADLHSC) from healthy donor using SPECT imaging after infusion through the portal vein in a patient with glycogenosis type 1A. Following infusion through the portal vein, SPECT imaging observed ADHLSC spread strictly within the targeted organ of liver until 5 days [55].

## 4. Theranostic Imaging of Stem Cells

The term “theranostics” refers to a rapidly developing approach that combines diagnostic imaging and molecular therapy [56]. By packaging the two modalities of diagnostic imaging and molecular therapy together, the application of theranostic imaging is promising to overcome undesirable selective biodistribution differences between diagnostic imaging probes and therapeutic agents [57–60]. To achieve higher sensitivity and increase molecular specificity, the novel two-in-one theranostic agents for various imaging tools have been developed. Not only can the novel imaging probes be functional as imaging tools, but they can also be useful as a carrier for controlled releasing of therapeutic moieties [61].

### 4.1. Improving Liver Function by Delivering Therapeutic Plasmid DNA

Pang et al. utilized a complex of SPIO nanoparticle coated with polyethylene glycol-grafted polyethylenimine (PEG-g-PEI-SPION) as an MRI probe that can track of rat bone MSCs and also act as a carrier for the plasmid DNA. The complex labeled MSCs were modified by a plasmid encoding human hepatocyte growth factor (HGF) attached in the MRI visible vector complex and transplanted into fibrotic rat livers. The results proved that these transplanted grafts effectively restored albumin production and significantly suppressed transaminase activities in the liver damaged animal models. Meanwhile, the transplanted MSCs displayed a sensitive signal on T2/T2^*∗*^-weighted MR images, which enabled in vivo tracking of the cells for up to 14 days after transplantation [62].

### 4.2. Promoting Transplanted Cell Survival by Combined Cytokines Delivery

Kempen et al. designed a theranostic mesoporous silica nanoparticle that could offer ultrasound and MRI signal to guide transplanted cells and also served as a drug release reservoir of insulin-like growth factor (IGF) that could promote MSCs survival. The results showed that the presence of IGF increased cell survival up to 40% versus unlabeled cells in vitro [63]. Pulavendran et al. compared cirrhotic mice received either hematopoietic stem cells (HSC) or MSC with or without HGF incorporated chitosan nanoparticles (HGF-CNP). Serum levels of selected liver protein and enzymes were significantly increased in the combination of MSC and HGF-CNP (MSC+HGF-CNP) treated group. These findings indicated that HGF-CNP enhanced the differentiation of stem cells into hepatocytes. The results demonstrated MSCs transplantation in combination with HGF-CNP could be a promising treatment for liver cirrhosis [64].

### 4.3. In Vivo RNA Interference: siRNAs and microRNAs Delivery Carriers

RNA interference is a naturally occurring endogenous regulatory process where short double-stranded RNA including small interference RNA (siRNA) and microRNAs could induce sequence-specific posttranscriptional gene silencing. In vivo RNA interference therapy represents a promising therapeutic strategy, but the barriers for delivery siRNAs or microRNAs hamper this therapeutic method to reach their intended targets cells or tissues and to exert their gene silencing activity [65]. Theranostic probes labeled on the transplanted stem cells actually could serve as siRNA or microRNA carriers. Zhao et al. synthesized poly-sodium4-styrenesulfonate (PSS) and poly-allylamine hydrochloride (PAH) coated AuNR-based nanoparticles, which could deliver siRNA against LSD1 to induce the hepatocyte lineage differentiation of human MSCs in vitro [66]. These methods need to be further tested in vivo before it could eventually translate to clinical applications. MicroRNAs are endogenous small noncoding RNAs that regulate key processes of cells. Studies have been reported on the posttranscriptional regulation of hepatic differentiation and regenerative capacities in MSCs by microRNAs [67, 68]. Gomes et al. reported the use of biodegradable nanoparticles containing perfluoro-1,5-crown ether (PFCE), a fluorine-based compound (NP170-PFCE) that could track cells in vivo by MRI and efficiently release miRNA. NP170-PFCE conjugated with miRNA132 could accumulate within the cell's endolysosomal compartment increased 3-fold the survival of endothelial cells (ECs) transplanted in vivo [69]. This novel theranostic technology potentially could be transferred to stem cells transplantation for liver diseases in future.

### 4.4. Stem Cells Can Be Used as a Unique Carrier for Therapeutic Agents

Zhao et al. loaded adipose-derived MSCs with SPIO-coated gold nanoparticles (SPIO@AuNPs), which is a MR contrast probe that can be activated to generate heat when irradiated with near-infrared laser, and tested their effects against liver injury and hepatocellular carcinoma (HCC) in mice. The results showed that in vivo MR imaging confirmed the active homing of AD-MSCs to liver. Upon laser irradiation, the SPIO@AuNP-loaded AD-MSCs could thermally ablate surrounding HCC tumor cells.

This study demonstrated that AD-MSC could act as an efficient carrier for therapeutic agents to liver injuries or HCC. The results showed that SPIO@AuNP–loaded AD-MSCs proved a promising theranostic agent for liver injury and HCC [70].

### 4.5. Preventing Teratoma Formation in Target Organs

One reason for controversial application of stem cell treatment is the possibility associated with tumor formation in the recipient. For promoting therapeutic effect, stem cells may undergo substantial manipulation such as differentiation and in vitro expansion, and this can lead to possible genetic aberrations and carcinogenesis [71]. To address the intractable issues such as the teratoma formation encountered in human embryonic stem cell (hESC) for therapeutic in clinic, Chung et al. hypothesized that serial manganese-enhanced MRI would have theranostic effect to assess hESC survival, teratoma formation, and hESC-derived teratoma reduction through intracellular accumulation of Mn2+. The study demonstrated that systemic administration of MnCl2 enabled simultaneous monitoring and elimination of hESC-derived teratoma cells by higher intracellular accumulation of Mn2+ [72].

## 5. Conclusions and Future Perspectives

Stem cell therapy as a part of regenerative medicine provides promising alternatives for the treatment of liver injuries and diseases. The increasing application of stem cells transplantation for liver diseases treatments created the demand for long-term and quantitative in vivo cell tracking methods [71]. In this review, promising in vivo imaging methods for stem cell monitoring after transplantation in liver are shown. It is unlikely that one technique will answer all questions, but use of a multimodal approach will be the most appropriate approach to addressing the number of issues posed in this exciting field. The selection of a set or a combination of appropriate imaging approaches will depend on the goal of the experiment, on the experimental subject under study, and also on the availability of multimodality imaging facilities and probes. In addition, new imaging modalities including Cherenkov illumination imaging (CLI), photoacoustic imaging (PAI), and surface enhanced Raman imaging (SERI) are developing rapidly. The application of these new strategies will also play a helpful role in the advancement of the field of stem cell therapy [73]. Mostly, although still in its infant stage of development, theranostic imaging definitely represents a promising new direction for in vivo stem cell transplantation. The maturation of multifunctional theranostic imaging may indeed revolutionize stem cell transplantation approach for liver diseases and beyond [74].

## References

[1] Schuppan D., Afdhal N. H. (2008). Liver cirrhosis. *The Lancet*.

[2] Berardis S., Sattwika P. D., Najimi M., Sokal E. M. (2015). Use of mesenchymal stem cells to treat liver fibrosis: current situation and future prospects. *World Journal of Gastroenterology*.

[3] Kuo T. K., Hung S.-P., Chuang C.-H. (2008). Stem cell therapy for liver disease: parameters governing the success of using bone marrow mesenchymal stem cells. *Gastroenterology*.

[4] Zhang Z., Wang F. S. (2013). Stem cell therapies for liver failure and cirrhosis. *Journal of Hepatology*.

[5] Lucey M. R., Terrault N., Ojo L. (2013). Long-term management of the successful adult liver transplant: 2012 practice guideline by the American Association for the Study of Liver Diseases and the American Society of Transplantation. *Liver Transplantation*.

[6] Wang P. P., Wang J. H., Yan Z. P. (2004). Expression of hepatocyte-like phenotypes in bone marrow stromal cells after HGF induction. *Biochemical and Biophysical Research Communications*.

[7] Luk J. M., Wang P. P., Lee C. K., Wang J. H., Fan S. T. (2005). Hepatic potential of bone marrow stromal cells: development of in vitro co-culture and intra-portal transplantation models. *Journal of Immunological Methods*.

[8] Volarevic V., Nurkovic J., Arsenijevic N., Stojkovic M. (2014). Concise review: therapeutic potential of mesenchymal stem cells for the treatment of acute liver failure and cirrhosis. *Stem Cells*.

[9] Amer M.-E. M., El-Sayed S. Z., El-Kheir W. A. (2011). Clinical and laboratory evaluation of patients with end-stage liver cell failure injected with bone marrow-derived hepatocyte-like cells. *European Journal of Gastroenterology and Hepatology*.

[10] Salama H., Zekri A.-R. N., Bahnassy A. A. (2010). Autologous CD34^+^ and CD133^+^ stem cells transplantation in patients with end stage liver disease. *World Journal of Gastroenterology*.

[11] Nikeghbalian S., Pournasr B., Aghdami N. (2011). Autologous transplantation of bone marrow-derived mononuclear and CD133^+^ cells in patients with decompensated cirrhosis. *Archives of Iranian Medicine*.

[12] El-Ansary M., Abdel-Aziz I., Mogawer S. (2012). Phase II trial: undifferentiated versus differentiated autologous mesenchymal stem cells transplantation in Egyptian patients with HCV induced liver cirrhosis. *Stem Cell Reviews and Reports*.

[13] Zekri A.-R. N., Salama H., Medhat E. (2015). The impact of repeated autologous infusion of haematopoietic stem cells in patients with liver insufficiency. *Stem Cell Research and Therapy*.

[14] Mohamadnejad M., Alimoghaddam K., Bagheri M. (2013). Randomized placebo-controlled trial of mesenchymal stem cell transplantation in decompensated cirrhosis. *Liver International*.

[15] Meier R. P. H., Müller Y. D., Morel P., Gonelle-Gispert C., Bühler L. H. (2013). Transplantation of mesenchymal stem cells for the treatment of liver diseases, is there enough evidence?. *Stem Cell Research*.

[16] Mohamadnejad M., Alimoghaddam K., Mohyeddin-Bonab M. (2007). Phase 1 trial of autologous bone marrow mesenchymal stem cell transplantation in patients with decompensated liver cirrhosis. *Archives of Iranian Medicine*.

[17] Zhang Z., Lin H., Shi M. (2012). Human umbilical cord mesenchymal stem cells improve liver function and ascites in decompensated liver cirrhosis patients. *Journal of Gastroenterology and Hepatology*.

[18] Peng L., Xie D.-Y., Lin B.-L. (2011). Autologous bone marrow mesenchymal stem cell transplantation in liver failure patients caused by hepatitis B: short-term and long-term outcomes. *Hepatology*.

[19] Salama H., Zekri A.-R. N., Medhat E. (2014). Peripheral vein infusion of autologous mesenchymal stem cells in Egyptian HCV-positive patients with end-stage liver disease. *Stem Cell Research and Therapy*.

[20] Xu L., Gong Y., Wang B. (2014). Randomized trial of autologous bone marrow mesenchymal stem cells transplantation for hepatitis B virus cirrhosis: regulation of Treg/Th17 cells. *Journal of Gastroenterology and Hepatology*.

[21] Shi M., Zhang Z., Xu R. (2012). Human mesenchymal stem cell transfusion is safe and improves liver function in acute-on-chronic liver failure patients. *Stem Cells Translational Medicine*.

[22] Vosough M., Moossavi S., Mardpour S. (2016). Repeated intraportal injection of mesenchymal stem cells in combination with pioglitazone in patients with compensated cirrhosis: a clinical report of two cases. *Archives of Iranian Medicine*.

[23] Pan X.-N., Zheng L.-Q., Lai X.-H. (2014). Bone marrow-derived mesenchymal stem cell therapy for decompensated liver cirrhosis: a meta-analysis. *World Journal of Gastroenterology*.

[24] Ma X.-R., Tang Y.-L., Xuan M., Chang Z., Wang X.-Y., Liang X.-H. (2015). Transplantation of autologous mesenchymal stem cells for end-stage liver cirrhosis: a meta-analysis based on seven controlled trials. *Gastroenterology Research and Practice*.

[25] Kim G., Eom Y. W., Baik S. K. (2015). Therapeutic effects of mesenchymal stem cells for patients with chronic liver diseases: systematic review and meta-analysis. *Journal of Korean Medical Science*.

[26] Than N. N., Tomlinson C. L., Haldar D., King A. L., Moore D., Newsome P. N. (2016). Clinical effectiveness of cell therapies in patients with chronic liver disease and acute-on-chronic liver failure: a systematic review protocol. *Systematic Reviews*.

[27] Yukawa H., Watanabe M., Kaji N. (2012). Monitoring transplanted adipose tissue-derived stem cells combined with heparin in the liver by fluorescence imaging using quantum dots. *Biomaterials*.

[28] Akhan E., Tuncel D., Akcali K. C. (2015). Nanoparticle labeling of bone marrow-derived rat mesenchymal stem cells: their use in differentiation and tracking. *BioMed Research International*.

[29] Wang C., Cheng L., Xu H., Liu Z. (2012). Towards whole-body imaging at the single cell level using ultra-sensitive stem cell labeling with oligo-arginine modified upconversion nanoparticles. *Biomaterials*.

[30] Li Z., Hu X., Mao J. (2015). Optimization of mesenchymal stem cells (MSCs) delivery dose and route in mice with acute liver injury by bioluminescence imaging. *Molecular Imaging and Biology*.

[31] Liu J. J., Hu X. J., Li Z. R. (2016). In Vivo bioluminescence imaging of transplanted mesenchymal stromal cells and their rejection mediated by intrahepatic NK cells. *Molecular Imaging and Biology*.

[32] Wu C.-G., Zhang J.-C., Xie C.-Q. (2015). In vivo tracking of human placenta derived mesenchymal stem cells in nude mice *via*
^14^C-TdR labeling. *BMC Biotechnology*.

[33] Bos C., Delmas Y., Desmoulière A. (2004). In vivo MR imaging of intravascularly injected magnetically labeled mesenchymal stem cells in rat kidney and liver. *Radiology*.

[34] Arbab A. S., Jordan E. K., Wilson L. B., Yocum G. T., Lewis B. K., Frank J. A. (2004). In vivo trafficking and targeted delivery of magnetically labeled stem cells. *Human Gene Therapy*.

[35] Cai J., Zhang X., Wang X., Li C., Liu G. (2008). In vivo MR imaging of magnetically labeled mesenchymal stem cells transplanted into rat liver through hepatic arterial injection. *Contrast Media & Molecular Imaging*.

[36] Ju S., Teng G.-J., Lu H. (2007). In vivo MR tracking of mesenchymal stem cells in rat liver after intrasplenic transplantation. *Radiology*.

[37] Ju S., Teng G.-J., Lu H. (2010). In vivo differentiation of magnetically labeled mesenchymal stem cells into hepatocytes for cell therapy to repair damaged liver. *Investigative Radiology*.

[38] Choi D., Kim J. H., Lim M. (2008). Hepatocyte-like cells from human mesenchymal stem cells engrafted in regenerating rat liver tracked with in vivo magnetic resonance imaging. *Tissue Engineering Part C: Methods*.

[39] Zhong Y., Tang Z., Xu R. (2013). Effect of transplantation route on stem cell migration to fibrotic liver ofrats via cellular magnetic resonance imaging. *Cytotherapy*.

[40] Kim T. H., Kim J. K., Shim W., Kim S. Y., Park T. J., Jung J. Y. (2010). Tracking of transplanted mesenchymal stem cells labeled with fluorescent magnetic nanoparticle in liver cirrhosis rat model with 3-T MRI. *Magnetic Resonance Imaging*.

[41] Shuai H.-L., Yan R.-L., Song H., Chen D.-L., Luo X. (2014). Analysis of feasibility of in vitro nuclear magnetic resonance tracking human umbilical cord mesenchymal stem cells by Gd-DTPA labeled. *Magnetic Resonance Imaging*.

[42] Shuai H., Shi C., Lan J., Chen D., Luo X. (2015). Double labelling of human umbilical cord mesenchymal stem cells with Gd-DTPA and PKH26 and the influence on biological characteristics of hUCMSCs. *International Journal of Experimental Pathology*.

[43] Pan H., Lan J., Luo X., Gao J., Xie X., Guo H. (2014). Biologic properties of gadolinium diethylenetriaminepentaacetic acid-labeled and PKH26-labeled human umbilical cord mesenchymal stromal cells. *Cytotherapy*.

[44] Ribot E. J., Gaudet J. M., Chen Y., Gilbert K. M., Foster P. J. (2014). In vivo MR detection of fluorine-labeled human MSC using the bSSFP sequence. *International Journal of Nanomedicine*.

[45] Ahrens E. T., Flores R., Xu H., Morel P. A. (2005). In vivo imaging platform for tracking immunotherapeutic cells. *Nature Biotechnology*.

[46] Helfer B. M., Balducci A., Nelson A. D. (2010). Functional assessment of human dendritic cells labeled for in vivo 19F magnetic resonance imaging cell tracking. *Cytotherapy*.

[47] Janjic J. M., Ahrens E. T. (2009). Fluorine-containing nanoemulsions for MRI cell tracking. *Wiley Interdisciplinary Reviews: Nanomedicine and Nanobiotechnology*.

[48] Chen J., Lanza G. M., Wickline S. A. (2010). Quantitative magnetic resonance fluorine imaging: today and tomorrow. *Wiley Interdisciplinary Reviews: Nanomedicine and Nanobiotechnology*.

[49] Zheng B., Vazin T., Goodwill P. W. (2015). Magnetic particle imaging tracks the long-term fate of in vivo neural cell implants with high image contrast. *Scientific Reports*.

[50] Zheng B., von See M. P., Yu E. (2016). Quantitative magnetic particle imaging monitors the transplantation, biodistribution, and clearance of stem cells in vivo. *Theranostics*.

[51] Ito-Fujishiro Y., Koie H., Shibata H. (2016). Tracking cells implanted into cynomolgus monkeys (*Macaca fascicularis*) using MRI. *Experimental Animals*.

[52] Shi X.-L., Gu J.-Y., Han B., Xu H.-Y., Fang L., Ding Y.-T. (2010). Magnetically labeled mesenchymal stem cells after autologous transplantation into acutely injured liver. *World Journal of Gastroenterology*.

[53] Spriet M., Hunt G. B., Walker N. J., Borjesson D. L. (2015). Scintigraphic tracking of mesenchymal stem cells after portal, systemic intravenous and splenic administration in healthy beagle dogs. *Veterinary Radiology and Ultrasound*.

[54] Gholamrezanezhad A., Mirpour S., Bagheri M. (2011). In vivo tracking of ^111^In-oxine labeled mesenchymal stem cells following infusion in patients with advanced cirrhosis. *Nuclear Medicine and Biology*.

[55] Defresne F., Tondreau T., Stéphenne X. (2014). Biodistribution of adult derived human liver stem cells following intraportal infusion in a 17-year-old patient with glycogenosis type 1A. *Nuclear Medicine and Biology*.

[56] Kelkar S. S., Reineke T. M. (2011). Theranostics: combining imaging and therapy. *Bioconjugate Chemistry*.

[57] Janib S. M., Moses A. S., MacKay J. A. (2010). Imaging and drug delivery using theranostic nanoparticles. *Advanced Drug Delivery Reviews*.

[58] Jokerst J. V., Gambhir S. S. (2011). Molecular imaging with theranostic nanoparticles. *Accounts of Chemical Research*.

[59] Ma X., Zhao Y., Liang X. J. (2011). Theranostic nanoparticles engineered for clinic and pharmaceutics. *Accounts of Chemical Research*.

[60] Wang P., Moore A. (2012). Theranostic magnetic resonance imaging of type 1 diabetes and pancreatic islet transplantation. *Quantitative Imaging in Medicine and Surgery*.

[61] Yoo D., Lee J.-H., Shin T.-H., Cheon J. (2011). Theranostic magnetic nanoparticles. *Accounts of Chemical Research*.

[62] Pang P., Wu C., Gong F. (2015). Nanovector for gene transfection and MR imaging of mesenchymal stem cells. *Journal of Biomedical Nanotechnology*.

[63] Kempen P. J., Greasley S., Parker K. A. (2015). Theranostic mesoporous silica nanoparticles biodegrade after pro-survival drug delivery and ultrasound/magnetic resonance imaging of stem cells. *Theranostics*.

[64] Pulavendran S., Rose C., Mandal A. B. (2011). Hepatocyte growth factor incorporated chitosan nanoparticles augment the differentiation of stem cell into hepatocytes for the recovery of liver cirrhosis in mice. *Journal of Nanobiotechnology*.

[65] Wang J., Lu Z., Wientjes M. G., Au J. L.-S. (2010). Delivery of siRNA therapeutics: barriers and carriers. *AAPS Journal*.

[66] Zhao X., Huang Q., Jin Y. (2015). Gold nanorod delivery of LSD1 siRNA induces human mesenchymal stem cell differentiation. *Materials Science and Engineering C*.

[67] Alizadeh E., Akbarzadeh A., Eslaminejad M. B. (2015). Up regulation of liver-enriched transcription factors HNF4a and HNF6 and liver-specific microRNA (miR-122) by inhibition of let-7b in mesenchymal stem cells. *Chemical Biology and Drug Design*.

[68] Chen K. D., Huang K. T., Lin C. C. (2016). MicroRNA-27b enhances the hepatic regenerative properties of adipose-derived mesenchymal stem cells. *Molecular Therapy. Nucleic Acids*.

[69] Gomes R. S. M., Neves R. P. D., Cochlin L. (2013). Efficient pro-survival/angiogenic miRNA delivery by an MRI-detectable nanomaterial. *ACS Nano*.

[70] Zhao J., Vykoukal J., Abdelsalam M. (2014). Stem cell-mediated delivery of SPIO-loaded gold nanoparticles for the theranosis of liver injury and hepatocellular carcinoma. *Nanotechnology*.

[71] von der Haar K., Lavrentieva A., Stahl F., Scheper T., Blume C. (2015). Lost signature: progress and failures in in vivo tracking of implanted stem cells. *Applied Microbiology and Biotechnology*.

[72] Chung J., Dash R., Kee K. (2012). Theranostic effect of serial manganese-enhanced magnetic resonance imaging of human embryonic stem cell derived teratoma. *Magnetic Resonance in Medicine*.

[73] Rodriguez-Porcel M. (2010). In vivo imaging and monitoring of transplanted stem cells: clinical applications. *Current Cardiology Reports*.

[74] Lee D. Y., Li K. C. P. (2011). Molecular theranostics: a primer for the imaging professional. *American Journal of Roentgenology*.

